# Early expression of neuroinflammation in an untreated fatal case of diabetic ketoacidosis

**DOI:** 10.20945/2359-4292-2024-0074

**Published:** 2024-10-04

**Authors:** Carol M. Artlett, Sabri H. Abdelwahab, William H. Hoffman, Ali S. Calikoglu

**Affiliations:** 1 Drexel University Drexel University College of Medicine Department of Microbiology and Immunology Philadelphia PA United States Department of Microbiology and Immunology, Drexel University College of Medicine, Drexel University, Philadelphia PA; 2 University of North Carolina Department of Genetics Chapel Hill NC United States Department of Genetics, University of North Carolina, Chapel Hill NC; 3 Augusta University Medical College of Georgia Section of Pediatric Endocrinology Augusta GA United States Section of Pediatric Endocrinology, Medical College of Georgia, Augusta University, Augusta, GA; 4 University of North Carolina Division of Pediatric Endocrinology Chapel Hill NC United States Division of Pediatric Endocrinology, University of North Carolina, Chapel Hill NC

## Abstract

We present the case of a young adult who had lethargy and significant weight loss for the three weeks before his death. The history of the present illness suggested a prodrome of several weeks, with progressive weakness indicating an advancing metabolic decompensation. To our knowledge, this is the first study performed on human brain tissue with type 1 diabetes (T1D) and likely diabetic ketoacidosis (DKA) before treatment. We studied neuroinflammatory markers in an insulin-deficient state without treatment compared with those found in a treated patient with T1D/DKA of similar age and race who died shortly after treatment. The frontal cortex and hippocampus were stained for tight junction proteins, RAGE, NLRP3, and HMGB1. Other markers that can disrupt the blood-brain barrier, such as IL-17, IL-6, IL-1β, GFAP, and IL-10 were also tested. This case study reveals that neuroinflammatory markers are expressed in the DKA brain at a lower level before treatment than those found to be expressed in the brain after treatment. These findings suggest that in DKA, dehydration minimizes inflammation which could be exacerbated with fluids promoting neuroinflammation and cognitive deficits. These findings require further studies and could identify therapeutic targets to reduce the progression of neuroinflammation and brain edema.

## INTRODUCTION

The systemic inflammatory response that is associated with acute illnesses is a very serious immunologic and metabolic complication in adults and children already seriously ill with type I diabetes (T1D)/diabetic ketoacidosis (DKA). The well-recognized DKA with hyperosmolar dehydration, metabolic acidosis, and serious electrolyte imbalances is also recognized as having diverse toxic inflammatory pathways. These dysregulations include both systemic and neuropathways that involve significant oxidative stress (1), advanced glycation end product/receptor for advanced glycation end product (AGE-RAGE) signaling (2), complement cascade (3), and the tryptophan/kynurenine pathway (4). While tight control of the systemic inflammatory response is protective (5–7), it can also lead to the progression of multiple organ failure and death (8).

Hoffman and cols. (9), reported sequential CT scans in children with T1D/DKA who had asymptomatic cerebrospinal fluid leakage due to subclinical brain edema. This occurred before the treatment of moderately severe DKA. We hypothesized that the pathogenesis of subclinical brain edema resulted from the dysregulated inflammatory prodrome, leading to a systemic inflammatory response (5,10). This observation piqued our interest in the early development and dysregulation of potential interactions that enhance neuroinflammation that precede the systemic inflammatory response (11). Our interest was further prompted by increasing reports of the early development of decreased cognition in newly diagnosed T1D/DKA in both children and adults (12–14). The prodrome of DKA is important since it becomes progressively toxic, and even more so with the formation of the systemic inflammatory response that follows briefly when intravenous treatment is begun (4,5,15). Interactions of various toxic pathways are definite possibilities such as with RAGE and quinolinic acid (16). Early pretreatment toxicity can result from slow resolution of previous episodes of suboptimal diabetic control if not fully resolved. Currently, there is limited information on the acute complications in humans with new-onset T1D regarding structural or neurohistopathological perturbations if not effectively treated.

Our studies were performed to compare neuroinflammatory markers in this untreated fatal DKA case with a treated fatal DKA case of similar age. Inflammatory biomarkers are rarely provided in admission laboratory tests. Our study sheds light on the understanding of the important interaction between dehydration/initial hydration and neuroinflammation.

## METHODS

Case history: A male, estimated to be in his early twenties, was received for autopsy. The subject displayed signs of dehydration with slight muscle wasting, measuring 177 cm in height and weighing 56 kg. There were no discernible needle marks, abrasions, bruises, or drug paraphernalia. The brain did not exhibit a clearly demarcated, inter-caudate margin, and there were no indications of ischemia or hemorrhages. Laboratory studies revealed a significantly elevated vitreous glucose level of 627 mg/dL. Toxicology and viral titers returned negative results. Despite numerous attempts, a comprehensive history could not be obtained due to unsuccessful endeavors to contact relatives or friends. Information from a neighbor indicated a period of three to four weeks of inactivity and weight loss. The subject was last seen being assisted into his apartment the evening before his death. An attempt to contact him the following morning found him in emesis, deceased in his small, sterile room. The control subject was a male, approximately the same age as the case, recently diagnosed (6 weeks) prior to death, who died shortly after correction of DKA. Both were coroners' cases, and brain tissues were submitted for analyses with anonymous coding.

Immunohistochemistry. The autopsies were performed at different institutions and with different autopsy protocols, however, the brain tissue was formalin-fixed and paraffin-embedded within 24 h and sectioned at 7 microns. The sections were deparaffinized with two changes of xylene and two changes of ethanol for 20 mins each, then processed to detect various proteins, see Table 1 for antibodies used and the staining conditions. If necessary, antigen retrieval was performed on the sections using 10 mM citrate buffer pH 6.0 in a microwave for 30 minutes, then washed with Tris-buffered saline before the quenching of peroxidases using 3% H_2_O_2_/methanol.

**Table 1:** Differences in biomarkers between the untreated fatal DKA and treated fatal DKA case

Biomarker	Untreated fatal DKA	Treated fatal DKA
NLRP3	0	0
RAGE	1-2	3-4
HMGB1	0	3
IL-1β	2-3	5
IL-6	2	4
IL-17	2-3	1
IL-10	0	0
GFAP	4	5
Fibrinogen	2	3
TJP (claudin and ZO-1)	3	0
TUNEL	0	3-4

0 = negative (no cells per 20X field of view); 1-2 weakly positive (1-10 cells per 20X field of view); 3-4 moderately positive (11-20 cells per 20X field of view); 5 strongly positive (>20 cells per 20X field of view).

Untreated case: total inflammatory score = 20-23. Less neuroinflammation Treated case: total inflammatory score = 28-30. Greater neuroinflammation.

Evaluations of staining were performed in the frontal cortex and hippocampus since these are sites of acute neuroinflammatory insults, residual deficits, and the foci of brain edema. Sections from these regions were stained for tight junction proteins of claudin-5 and zonula occluding-1 and examined for structural alterations. Biomarkers of neuroinflammation such as RAGE Cat#PA5-2478 (3,15), NLR family pyrin domain containing 3 (NLRP3, Cat#PA5-79740) (17), and high mobility group box 1 (HMGB1, Cat#MA5-31967) (18). Briefly, the staining procedure for these antibodies included antigen retrieval in 10 mM citrate buffer pH 6.0 in a microwave for 30 minutes, sections washed in PBS, and then blocked with 5% donkey serum. The antibodies were diluted 1:50 and applied to the sections overnight at 4 °C, washed in three changes of PBS, and then donkey-anti-rabbit-HRP applied for 4 h. The sections were washed and the bound HRP reacted with HRP Conjugate Substrate Kit Bio-Rad Cat#1706431. The remaining markers with the potential to disrupt the blood-brain barrier include interleukin (IL)-17 (19), IL-6 (20), IL-1β (20), glial fibrillary acidic protein (GFAP) (21), and the anti-inflammatory biomarker IL-10 (6) were investigated. These antibodies have been previously reported (20). Apoptosis was tested using terminal deoxynucleotidyl transferase biotin-dUTP nick end labeling (TUNEL). The TUNEL kit was purchased from Abcam, Waltham MA, and tissues were stained according to the protocol supplied in the kit without modification.

The semiquantitative score used was an estimated number scored from 1 to 5 done in a masked fashion by two graduate students and WHH each scoring three 3 fields at 20X magnification, which were then averaged. CMA scored ten random fields of view for NLRP3, RAGE, HMGB1, and TUNEL at 20X magnification which were also averaged. Fields of view were classified as being negative (0) with no cells per 20X field of view, weakly positive (1–2) with 1-10 cells per 20X field of view, moderately positive (3–4) with 11-20 cells per 20X field of view, or strongly positive with greater than 20 cells per 20X field of view.

In both cases presented here, the brain assessed for inflammatory biomarkers were derived from the remaining tissues once the autopsy was completed. We received them deidentified of their autopsy number. Because these were tissues received from cadavers, Federal Regulations 45 CFR 46, does not consider this to be Human Subjects research.

## RESULTS

**Figure 1:**
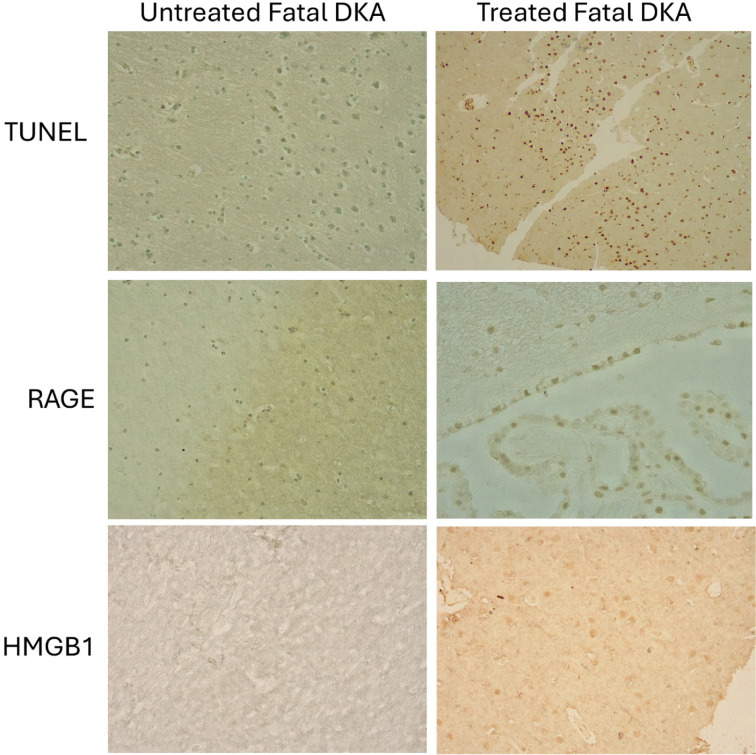
Comparison of Inflammatory Markers Between Treated and Untreated Cases of DKA. Example stains of TUNEL, RAGE, and HMGB1 between the two cases show the lesser extent of inflammatory markers in the untreated case compared to the greater elevation of inflammatory markers in the treated case. All images were photographed at 20X magnification.

Table 1 summarizes a consistent pattern of fewer and/or absent biomarkers in the untreated versus treated fatal T1D/DKA case. The striking lesser expressions of RAGE and HMGB1 (Figure 1) in the untreated case compared to the treated case are also present for the early and usually more prevalent inflammatory cytokines in DKA, namely IL-6, and IL-1β. TUNEL is highly elevated in the treated DKA case and absent in the untreated case (Figure 1). Of interest is the absence of NLRP3 expression in both cases.

## DISCUSSION

The subclinical brain edema of DKA is likely caused by the background level of inflammatory biomarkers in the prodrome before treatment (22). Shortly after the initiation of treatment, there is an immediate increase in the blood flow and the prodrome (23) with a rapid formation of toxic alpha dicarbonyls (15) along with the rapid formation of the systemic inflammatory response.

Admission laboratory results only provide information on acidosis, dehydration, and metabolic dysregulation, but not the toxic inflammatory biomarker candidates in the brain. This lack of early potential toxic markers could be targeted for blunting or ablation of advancing neuroinflammation and possibly the crisis of clinical brain edema. Our comparison of these two cases allows for the opportunity to address the important question that Brown raised twenty years ago (24) in his paper entitled "Cerebral oedema in childhood diabetic ketoacidosis: is treatment a factor?" and to date this has not been answered. The early progressing inflammatory prodrome becomes the sudden expression of the systemic inflammatory response that is initiated at the time of hydration (5), and the increased blood flow (23). Our data (4,5,15) reports that rehydration of DKA enhances the expression of numerous neurotoxins including inflammatory cytokines (4,6,7) and the dicarbonyl ligands (15) for the AGE-RAGE pathway (2,25). We hypothesize that the inflammatory neuronal perturbations likely result in the cognitive deficits reported in children and adults with T1D/DKA (12–14).

The relationship between dehydration and immune inflammation has received limited attention however, a recent study of healthy dehydrated adults challenged with lipopolysaccharide showed a blunted inflammatory response (26). This supports our findings that in DKA dehydration minimizes inflammation, while hydration promotes the development of the systemic inflammatory response (5,15), which is then gradually suppressed by the intravenous insulin that accompanies hydration. Lack of hydration would result in a very gradual if any, increase in inflammation of systemic (15) or neurological (5) in a DKA crisis. The increased expression of neuroinflammation (gliosis) was also reported by Lo and cols., in an insulin-deficient rodent model (27) and also supports our earlier findings in humans that the initiation of hydration in DKA intensifies inflammation. This is prior to the gradual suppression of inflammation as treatment ensues with insulin (5).

Previously reported is that systemic RAGE levels are increased after DKA treatment (25). In our untreated case, RAGE levels in the brain were expressed albeit at lower levels than in the treated fatal DKA case (Figure 1). This difference of less perturbation was similar to the other inflammatory markers tested. In contrast, HMGB1 was not expressed in the untreated case but was expressed in the treated case (Table 1, Figure 1). This too supports that hydration mediates and enhances inflammation in our treated case (2,5,27). In addition to RAGE's and HMGB1's decreased/absent expression, there was also a lower expression of the early and more prevalent DKA cytokines, IL-1β, and IL-6 (5) in the frontal cortex and hippocampus in the untreated case (Table 1).

The heterogeneity of astrocytic glial cells, with their significant plasticity, emphasizes the recognition of the complicated DKA milieu influencing astrocytes in their role as mediators. We have confirmed a dramatic increase in the activity of GFAP (2), a cytoskeletal constituent similar to changes reported by Hawrylak and cols. (28). We also identified the association of GFAP with astrocytosis and its colocalization with RAGE and the known autocrine loop that propagates reactive gliosis in this fatal DKA case.

The absence of activation of the NLRP3 inflammasome but the modest expression of IL-1β in the untreated case is an interesting observation. This could be due to the suppressive effect of β-hydroxybutyrate (29) and the release of IL-1β could be via non-canonical activation of caspase-4 and caspase-5 (30). This could cause pyroptosis or inflammatory cell death in the absence of apoptosis as in the TUNEL-negative untreated patient, while the treated patient had elevated TUNEL likely the result of the increased inflammation during treatment (Figure 1).

While this comparison study is important and we have drawn conclusions, our study has some limitations. One of these limitations is the lack of clinical history in the untreated case. The history of this case was obtained from the neighbor. Conclusions of DKA were drawn from the neighbor's statement pointing to approximately four weeks of inactivity, weight loss, and significant weakness the prior evening. He was found the next morning deceased in vomit. At autopsy, it was noted that he was dehydrated with muscle wasting and his vitreous glucose levels were 627 mg/dL. All these findings are strongly suggestive of DKA.

Another caveat to these studies is that we cannot entirely rule out the impact of proteolysis, enzymatic degradation, or autolysis affecting the overall assessment of the inflammatory biomarkers in the brain. The length of the post-mortem interval cannot be ruled out and this is always a challenge when studying autopsy materials. However, it should be noted that these degradative changes in the brain do not always decrease antigenicity. For example, proteolysis was found to increase GFAP detection (31,32). In both cases, the autopsy was performed within 24 hours.

Extended prodromes and increasing dehydration are likely to cause more severe neurocognitive deficits. Also, aggressive hydration can cause an increased release of inflammatory molecules that can further compromise neurocognition. Serious consideration must be given to the delay in treatment as well as the aggressiveness of hydration. While the latter must be addressed this might not enhance the well-recognized earlier decrease in neurocognition. A focused admission history is vital to be able to perceive the impending neurocognitive impact of an inflammatory prodrome. The decision to administer fluids on admission requires a carefully focused history of the gastrointestinal and neurological symptoms and signs that would influence the treatment decision rather than the use of a set protocol. There is a sense of urgency in both the laboratory and bedside to recognize the likelihood of pretreatment cognitive insults worsening after the initiation of hydration and the increase in DKA's systemic inflammatory response.

Here we show that in the untreated case, there is the presence of neuroinflammation prior to the treatment for DKA and that this level of inflammation can increase with treatment. However, we recognize cognitive perturbations can also remain static, regress, or progress, and this requires more extensive study. These findings on the early toxic biomarkers of neuroinflammation point to novel therapeutic targets that could be used to blunt or ablate the advancing neuroinflammation and possibly the crisis of clinical brain edema.
